# Perceived Needs and Barriers in Raising a Child with ASD and Accessing Early Interventions in Qatar: Parents’ Voices

**DOI:** 10.2147/JMDH.S525521

**Published:** 2025-09-09

**Authors:** Lara M Saranek, Mujahed Shraim, Nazim Abdel Aati, Ghadir Fakhri Al-Jayyousi

**Affiliations:** 1 Department of Public Health, College of Health Sciences, QU Health Sector, Qatar University, Doha, Qatar; 2 Developmental Behavioral Pediatrics, International Child Development Centers, Doha, Qatar

**Keywords:** autism spectrum disorder, ASD service, parent perceptions, barriers, mental health

## Abstract

**Background:**

Autism Spectrum Disorder (ASD) is a lifelong neurodevelopmental disorder that is often associated with mental health comorbidities. Parents are challenged with raising a child diagnosed with ASD. However, research about parental challenges in Arab countries is limited. The study aims to explore parents’ experiences of raising a child with ASD and accessing early interventions provided for their children in Qatar.

**Methods:**

An inductive qualitative study design was applied. Data was collected using face-to-face interviews with parents (n=20). Interviews were transcribed verbatim, and thematic analysis was employed.

**Results:**

The main elicited themes from the analysis were mainly about: challenges of raising a child diagnosed with ASD; parents’ perception of existing ASD services; parents’ perception of mental health services for children diagnosed with ASD; parents’ perception of the impact of the COVID-19 pandemic on ASD services; and the impact of raising a child diagnosed with ASD on parents.

**Conclusion:**

Parents face challenges with raising a child diagnosed with ASD and with accessing ASD early intervention services for their children. The findings guide health care professionals working with children with ASD and their families, and policy makers in developing standards of care for ASD service in the governmental facilities in Qatar.

## Background

Autism Spectrum Disorder (ASD) is a lifelong neurodevelopmental disorder characterized by symptoms related to persistent deficits in social communication and interaction, including deficits in social reciprocity, nonverbal communication, and skills required for developing, maintaining, and understanding relationships.^1^ The cause of ASD remains unknown.^2^ However, the etiology of ASD is likely to be multifactorial, including genetic and non-genetic risk factors.^2^ ASD is often associated with mental health comorbidities;^3^ therefore, the management of ASD requires an early comprehensive multidisciplinary approach and long-term follow-up, which is often individualized according to the severity of the conditions and associated comorbidities.

According to the World Health Organization (WHO), about 1 in 100 children is diagnosed with ASD.^4^ In the Middle East, a review showed that ASD prevalence was; 1.6 per 1000 children and adolescents aged 6–18 years in Iran, 1.4 per 10,000 children and adolescents aged 0–14 years in Oman (95% CI 1.2, 1.7), 11.4 per 1000 children and adolescents aged 6–11 years in Qatar 1.14% (95% CI 0.89, 1.46).^5^ Supporting an individual with ASD over the lifespan is associated with several challenges at different levels.^6^

Parents face challenges related to accessing appropriate healthcare services for their children with ASD.^7–10^ A study showed that caregivers were first concerned about their child’s development at the age of 2.7 years, while the ASD diagnosis was made at an average age of 5.5 years.^11^

### Parental Challenges in Arab Countries

Research examining parental challenges with ASD in Arab countries is limited. Jordanian parents reported issues related to the availability of ASD services, having to rely on multiple ASD service providers to receive care for their child, the services’ financial burden, and the parent-to-provider collaboration level.^12^ A study in Qatar reported parental dissatisfaction with long waiting to receive ASD services, and health-related quality of life scores were significantly low among parents of children diagnosed with ASD.^13^

### The National Autism Plan and ASD Early Intervention in Qatar

The Qatar National Autism Plan was developed in 2017 based on recommendations from ASD experts at the WHO.^14^ The plan aims to improve the lives of individuals with ASD and their families by addressing a range of issues, including awareness, early recognition and screening, diagnosis and assessment, interventions, education, and the transition into adolescence, adulthood, and old hood.^15^ Only one governmental center offers ASD early intervention in Qatar, which provides speech therapy, occupational therapy, group-based special education for school enrollment preparation, all based on Applied-Behavior Analysis (ABA) principles, and the parent support program EarlyBird (EB). The duration of the early intervention program is 3 months, with a frequency of once weekly to once every two weeks for each of the interventions provided. In the present study, we explore parents’ experiences of raising a child with ASD and accessing early interventions provided for their children in Qatar.

## Methods

### Aim

The purpose of this study is to understand the experience of raising a child with ASD and accessing early interventions provided for children in Qatar from parents’ perspectives.

### Research Study Design

Qualitative research provides insights and understanding of individuals’ experiences and is used to understand facilitators and barriers to successful intervention implementation.^16^ In this study, we followed an inductive qualitative approach in which the researcher is not occupied or biased with predetermined assumptions and is open to understanding a new phenomenon to answer the research question.^17^,^18^

### Study Setting

The study was conducted in the governmental hospital in Qatar. The hospital provides specialized child development services with mental and physical disabilities, developmental and learning delays 19 The center consists of five specialized multidisciplinary clinics, including the ASD early intervention program, disability support program for students, specialized services such as the pediatric assistive technology services, and pediatric psychology services.^19^ Study participants were parents of children diagnosed with ASD (n = 20), aged less than 6 years, attending ASD early intervention for their children at the governmental hospital in Qatar. Parents were invited to participate in the interview during follow-up visits to the hospital for their children. The clinical nurse asked parents while they were waiting to be called for their appointments in the hospital outpatient clinics’ waiting areas. The nurse provided the parent with an overview of the research and obtained initial verbal consent to participate in the study. If the parent consents, the clinical nurse introduces the researcher to the parent. Next, the clinical nurse, the researcher, and the participant arranged a specific time during the day for the interview at the participant’s convenience. At the time of the interview, the researcher and the participants are guided to a private designated area in the hospital (the center’s meeting room and non-utilized consultation room) to maintain privacy. Since talking about challenges may be upsetting, and parents would get emotional, especially since ASD and mental health conditions are still considered stigmatized in the region. In the interview room, the researcher introduces herself again, explains the purpose of the research, and reassures the participant that participating in the study is voluntary. The researcher provided the study information sheet and informed consent form in the participant’s preferred language, gave parents the time to read the forms, and sign them upon their consent. Next, in-depth, face-to-face, semi-structured interviews were conducted with parents in the designated private area in the center, for a profound and comprehensive address of the study aim. The interviews were conducted between October 2021 and November 2021.

### Participants, Sampling, and Data Collection

This study was approved by the Institutional Review Board at the university (1625-EA/21) and by the Governmental Corporation (MRC-01-21-406). Study participants were selected using a convenience sampling approach. Participants are: (1) parents of children diagnosed with ASD who are aged under 6 years; and (2) attending ASD early intervention for their children at the governmental hospital in Qatar. To broaden our understanding of parental experiences, we tried to recruit mothers and fathers of different nationalities, educational levels, and employment statuses, which provided a rich understanding of the phenomenon. Parents were invited to take part in the qualitative interviews while attending follow-up visits in the Child Development Center for their children. The clinical nurse asked parents to participate in the study while they were waiting to be called for their appointment in the waiting areas of the CDC outpatient clinics. The nurse provided the parent with an overview of the research and obtained initial verbal consent to participate in the study. If the parent consent, the clinical nurse would introduce the researcher to the parent. Next, the clinical nurse, the researcher, and the participant would arrange a specific time for the interview at the participant’s convenience. At the time of the interview, the researcher and the participants guided to a private designated area in the CDC (the center’s meeting room and non-utilized consultationroom), to maintain privacy, so parents would not feel embarrassed in expressing their emotions. Talking about challenges may be upsetting, and parents would get emotional, especially since ASD and mental health conditions are still stigmatized in the region. In the interview room, the researcher would introduce herself again, explain the purpose of the research, and reassure the participant that participating in the study is voluntary. The researcher would provide the study information sheet and consent form in the participant’s preferred language, give parents the time to read the forms, and sign them upon their’ consent. Face-to-face, semi-structured interviews were conducted to capture parental experiences and perceptions of raising a child diagnosed with ASD and ASD service access. Individual interviews are useful in capturing an in-depth understanding of the phenomenon, maximizing the quality of collected data, and capturing sensitive data.^20^ Interviews were conducted in Arabic & English according to the participants’ preferences, and were audio-recorded. Each interview lasted approximately 45 minutes.

After reviewing the literature about parental experiences with ASD early intervention and the related mental health services, we constructed the interview guide (See Appendix 1). Interview questions were originally written in English and then translated into Arabic by the first author and validated by the other authors. Interview questions were constructed to capture parents’ experience of accessing and receiving ASD early intervention, and their perception of mental health services for their child. Upon parents’ responses, probes were demonstrated to obtain additional information and to get an in-depth understanding of parents’ experiences. The interview started with demographic questions, then there was a set of questions about parental views on challenges with raising a child with ASD and on accessing the existing ASD service. Data collection continued until data saturation was reached and an in-depth understanding of the study aim was obtained, and no further new themes emerged.^21^ Data saturation was reached when there was enough replicated information, and the ability to obtain additional information was attained.^22^

### Data Analysis

Inductive thematic analysis was followed to discover and elicit the major themes in the data. An inductive approach was employed in which the researcher is not occupied with predetermined assumptions and will be open to any emergent themes that will help understand the phenomenon and answer our research question.^17^,^18^ Thematic analysis encompasses identifying, analyzing, and interpreting patterns of meaning within the qualitative data to answer the research question.^17^,^18^ Data collection continued until data saturation was reached and an in-depth understanding of the study aim was obtained, and no further new themes emerged.^21^ Data saturation was reached when there was enough replicated information, and the ability to obtain additional information was attained, and further coding was no longer feasible.^22^ Manual data analysis was employed using colors and numbers during the coding process to highlight the themes and subthemes. Data analysis was conducted with the transcribed interviews in their original languages, to ensure capturing the right meaning and maintaining the validity of experiences. The analysis process started after conducting and transcribing the first interview. The first step in the analysis was coding the first interview to construct our codebook, which consisted of the emergent themes and subthemes. In alignment with the study aim, multiple reading practices supported the coding processes and generated the study themes. The constructed codebook was used by the PI and Co-PI to code the remaining transcripts, and new themes were added to the codebook as they emerged during the data analysis process. To enhance inter-coder reliability, the PI and Co-PI (two researchers) coded the first interview blindly and independently, then they met to discuss the discovered themes and resolve any disagreements. We applied triangulation in the analysis process to enhance the research credibility. In the triangulation process, we involved two researchers to observe, process, and analyze data separately, to improve the credibility of reported findings. The researchers listened to the recorded interviews separately. We first employed open coding, and then axial coding. During the open coding process, we highlighted all statements that help answer our research question. The open coding process involved breaking data into discrete parts, by continuously comparing data to identify similarities and differences and collate similar pieces of data, then labeling them into codes with each code labeled using a single color and a unique number (eg, code no. 2.00). In the open coding process, codes were adjusted, rephrased, and evolved over the coding process until final refined codes were developed. The second level of coding was axial coding, which involved identifying relationships between codes for the purpose of developing categories.^23^ During axial coding, we drew connections between developed codes in the open coding step, then codes were grouped as categories, and accordingly, themes were developed.

## Results

### Participants’ Characteristics

Study participants were parents of children diagnosed with ASD attending an ASD early intervention service in the governmental hospital (n = 20; Mothers = 13, Fathers = 6, and Caregivers = 1). They were of different nationalities, including Qatari, Tunisian, Palestinian, Syrian, Filipino, Sri Lankan, Pakistani, and Indian. Among the interviewed parents, six parents have male children diagnosed with ASD, fifteen parents have female children, and one parent has both male and female children diagnosed with ASD. See Table 1 for participants’ characteristics.

**Table 1:** Demographic Characteristics of Interviewed Parents (n=20)

Variable	Number	Percentage
Age^a^	36	9
Relationship to child		
*Mother*	14	70.0
*Father*	5	25.0
*Caregiver*	1	5.0
Nationality		
*Qatari*	4	20.0
*Other Arab nationalities*	6	30.0
*Other nationalities*	10	50.0
Marital Status		
*Married*	19	95.0
*Divorced*	1	5.0
Education Level		
*Secondary school*	5	25.0
*Diploma or Bachelor's*	11	55.0
*Master’s or PhD*	4	20.0
Employment Status		
*Full time*	13	65
*Unemployed*	7	35

**Notes**: ^a^Variable with numeric scale is reported as median and interquartile range. Italic text refers to sub-group under main variable category.

### ASD Service Access Trajectory

The journey of ASD service access started with a trigger to seek ASD services, followed by parents’ response to ASD diagnosis, parents’ perception of ASD etiology, and finally accepting Autism. The triggers were mainly about the child's symptoms observed by the parent, which included speech delay, social disengagement, deficits in social communication and interaction, difficulty in expressing the child’s self, lack of eye contact and attention, self-harm, hypersensitivity and agitation from loud noise, sensitivity to certain food textures, reduced follow of instructions, and hiding and sitting alone. Other identified triggers include comparison with other children, watching ASD awareness videos, looking up ASD symptoms, and available services online. For some parents (n=7), the most difficult time was the time of breaking the news of the ASD diagnosis. ASD journey encompassed self-blame for some parents and self-education (n=7). A few parents (n=2) had misconceptions about ASD etiology. Eventually, parents adapted, coped, and accepted their child's diagnosis.

Elicited themes from the analysis were mainly about: challenges related to raising a child diagnosed with ASD; parents’ perception of existing ASD service; parents’ perception of mental health services for children diagnosed with ASD; parents’ perception of the impact of the COVID-19 pandemic on ASD service; and the impact of raising a child diagnosed with ASD on parents. See Table 2 for themes’ dispersion.

**Table 2:** Themes Dispersion

Theme 1	Challenges Related to Raising a Child Diagnosed with ASD	Parents n (%)	Quotes
Subthemes	Parent-child communication	7 (35)	“I don’t what is wrong with him, or if he is not alright, I know one gesture meaning he is irritated, but he can’t tell me what he is feeling, I don’t know what hurts him” (Participant. 9).
	Severity of the disorder	6 (30)	“He is a very I would say a stubborn, very stubborn, if he does not want to pick things up, he would not pick things up, but the therapists are very patient with him I am not complaining about the therapists, but him in general child he is a very moody child, and he is not okay with new people touching him around” (Participant. 18).
	Family, society & social stigma	6 (30)	“Nobody understands in the family not anyone accepts that this is Autism, there are two concepts for ASD for them, either sorry to say the child is crazy, loss his/her recognition or impolite and needs to be set to be polite” (Participant. 7).
	Child’s acceptability of the service	4 (20)	“It is not a challenge it is about the mood, if he is today in the mood all goes easy, see today he is not in the mood, although he slept early. Sometimes his lack of concentration, he ignores when the specialist talks to him, you see him looking at something else, but as I said at the end it turns out it is his mood” (Participant. 13)
	Parental concern about child’s future	5 (25)	“Yes, overthinking what will happen in the future will he be normal afterwards; you don’t know actually so that is the main challenge of having an Autistic child” (Participant. 15)
**Theme 2**	**Parents’ Perception of Existing ASD Service**		
Subthemes	Delay in receiving ASD service	15 (75)	“Still one year to get first appointment, we came here he was 2.5 years to get first appointment later the next session they said after one year, during COVID it is difficult” (Participant. 12).
	Service design, intensity & duration	12 (60)	“You know children diagnosed with ASD particularly, those who are really sick more than expected, the severe type, will fear, if the Dr sees the child once weekly, the child will fear and he/ she will not like the Dr so it should be more than one day per week so the child himself/ herself get used to the doctor so he/she can get along with the doctor so he/ she can learn, for example if the doctor wants him/ her to speak, the child will feel shy to do so, he/ she will not be used to the doctors, so they will not speak, that’s why” (Participant. 3)
**Theme 3**	**Parents’ perception of mental health services for children diagnosed with ASD**		
Subthemes	Parents’ perception of the need to access the mental health services	18 (90)	“I think Autism is not a mental problem, Autism is just a developmental delay and Autism makes every child different, but it does not mean your child is mentally challenged in any way, you just need to address the issues that they have whether it is sensory, whether it is speech, whether they have problems expressing themselves, I do not see Autism as mental challenge” (Participant. 18)
	Parents’ perception of the effectiveness of the mental health services	5 (25)	“There is no change, no improvement at the end, follow up must be weekly, and it should be daily if needed, if they provide us a center, to give daily sessions for children” (Participant. 7).
	Parents’ misconceptions	6 (30)	“For me, mental health is something that I never associated that part, so I never thought about it to be very honest” (Participant. 10).
**Theme 4**	**Parental Perception of the Impact of COVID-19 on ASD Service**	11 (55)	“I did not find a way out. The parks and malls were closed. He was at the beginning of his awareness to know these things. Places close-up was the reason my son’s case got worse. Corona was a difficult period. All these factors led to decline in his progress but thank God I was trying with him” (Participant. 8)
**Theme 5**	**Impact of Raising a Child Diagnosed with ASD on Parents**		
Subthemes	Personal wellbeing of parents of children diagnosed with ASD.	9 (45)	“I don’t know what hurts him at night, I don’t sleep, just because I fear that something would happen to him at night**”** (Participant. 9)
	Mental wellbeing of parents of children diagnosed with ASD	12 (60)	“The experience of having a child with ASD is more difficult than what the human mind can imagine, it needs you to be alert and awake like in the military, 24 hours 7 days a week, you never have time to rest. At the end we are human beings” (Participant. 7). “Because you will not have that much peace of mind you know, when your child is growing up, will he be normal afterwards? Will he be like this on that time? You know, so it is more an emotional challenge, if he is normal child, we will just see him play sitting down so yes, more of emotional actually” (Participant. 15)
	Financial Impact	7 (35)	“The financial side, outside the session that lasts less than an hour could reach QAR 400–500, one session is not enough, you find yourself monthly paying not less than QAR 5000–6000, which is out of budget for the majority” (Participant. 7)

**Note**: Bold text refers to main theme.

### Theme 1: Challenges Related to Raising a Child Diagnosed with ASD

Challenges about raising a child with ASD were parent-to-child communication, severity of the disease, family, society, and social stigma, child’s acceptability to the service, and parental concern about the child’s future.

#### Subtheme: Parent-Child Communication

Miscommunication between parents and their autistic children is a highlighted challenge. The child diagnosed with ASD is unable to express his/her needs, anger, or agitation, not even to their parents. Parents indicated that this barrier limits their ability to understand their child’s needs, demands, and source of pain.

I don’t know what is wrong with him, or if he is not alright. I know one gesture means he is irritated, but he can’t tell me what he is feeling, and I don’t know what hurts him. (Participant 9)

#### Subtheme: Severity of the Disease

Interviewees had concerns related to the child’s clinical manifestations, behavior, and development due to the diagnosis. These concerns include the child’s start time to speak. Additionally, parents were distressed with the child’s behaviors, including anger, hysteria, stubbornness, moodiness, aggression, lack of concentration and responsiveness with the parent, high focus with specific toys only, obsession with certain interests, social disengagement, physical inactivity, and the child’s reduced awareness about the surrounding environmental risks.

He is a very I would say a stubborn very stubborn. If he doesn’t want to pick things up, he wouldn’t pick things up, but the therapists are very patient with him. I am not complaining about the therapists, but him in general child he is a very moody child, and he is not okay with new people touching him around. (Participant. 18)

#### Subtheme: Family, Society & Social Stigma

Relatives’ and friends’ perceptions were an encountered challenge by parents, as in some cases, the child’s diagnosis is not accepted by the family, where the community views the child as intellectually challenged or impolite. Parents were significantly concerned about their child’s behavior in public. Social gatherings were perceived by parents as a burden due to frequent questions about the child’s behavior and constant judgment. Another concern was the label of having an ASD record in the hospital setting.

Nobody understands in the family not anyone accepts that this is Autism, there are two concepts for ASD for them, either sorry to say the child is crazy, loss his/her recognition or impolite and needs to be set to be polite. (Participant. 7)

#### Subtheme: Child’s Acceptability of the Service

While receiving ASD intervention, children-based challenges observed by parents included hyperactivity, lack of cooperation with professionals, the child is not encouraged to receive the service for mood reasons, lack of concentration, stubbornness, and hypersensitivity. One case has encountered Post-Traumatic Stress Disorder (PTSD) from hospitals, the child had a bad recall experience from an earlier medical procedure, and this by means acts as a major challenge in receiving ASD intervention.

It is not a challenge it is about the mood, if he is today in the mood all goes easy, see today he is not in the mood, although he slept early. Sometimes his lack of concentration, he ignores when the specialist talks to him, you see him looking at something else, but as I said at the end it turns out it is his mood. (Participant. 13)

#### Subtheme: Parental Concern About Child’s Future

One raised concern by parents is the child’s future in terms of educational qualifications for a typical schooling experience, as well as the documented education type (as special needs), and the potential of not qualifying to enroll in college and obtain a career accordingly. Parents exhibited worrying about independence in adulthood, obtaining education and career, getting married, and overall self-development. Parents were highly concerned about the stage when parents are no longer expected to support their sons/daughters, or after the parents’ death.

Yes, overthinking what will happen in the future will he be normal afterwards; you don’t know actually so that is the main challenge of having an Autistic child. (Participant. 15)

### Theme 2: Parents’ Perception of Existing ASD Service

Parental perception of accessing ASD service and the related barriers were explored, arising subthemes were delay in receiving ASD service, service design, program intensity, and duration.

#### Subtheme: Delay in Receiving ASD Service

Most parents raised concerns regarding the waiting time to receive an assessment for an ASD diagnosis. As perceived by parents, the referral pathway of ASD suspected cases from primary care to the governmental hospital is not clear. Parental perceived factor of delayed service includes reduced workforce capacity since this center is the only facility in Qatar. Overall, the time delay in receiving ASD early intervention contributed to having a poorer child’s condition, and the more the time passes while on the waiting list, the more the symptoms and behaviors are difficult to manage, parents highlighted. Thus, parents sought services from the private sector for diagnosis and early intervention.

Still one year to get first appointment, we came here he was 2.5 years to get first appointment later the next session they said after one year, during COVID it is difficult. (Participant. 12)

#### Subtheme: Service Design, Intensity, and Duration

Parents were concerned about the inflexible procedure followed in the governmental healthcare setting. They stated that the clinical management plan should be customized depending on each case’s needs. Another concern was the lack of applicability of the provided intervention at home. According to parents, this center is the only one, and the traveling distance to the center is considered long. Parents showed high dissatisfaction with the program’s low intensity since it is provided once weekly or once every two weeks over a period of 3 months; thus, it is almost ineffective with this intensity. Additionally, the overall duration of the program was perceived as short, and the program should be longer for a substantial impact on the child’s improvement. On the other hand, a few parents showed satisfaction with the service quality in the governmental hospital. They stated that the team is very specialized, professional, cooperative, supportive, and that children receiving the intervention were happy with the team. Parents shared positive feedback about the parent teaching program EarlyBird; they found it highly relevant and beneficial. Although parents had access to private centers, they perceived the governmental service as more credible and trustworthy.

You know children diagnosed with ASD particularly, those who are really sick more than expected, the severe type, will fear, if the Dr sees the child once weekly, the child will fear and he/ she won’t like the Dr so it should be more than one day per week so the child himself/ herself get used to the doctor so he/she can get along with the doctor so he/ she can learn, for example if the doctor wants him/ her to speak, the child will feel shy to do so, he/ she will not be used to the doctors, so they won’t speak, that’s why. (Participant 3)

### Theme 3: Parents’ Perception of Mental Health Services for Children Diagnosed with ASD

Parents’ perceptions of mental health services for their children were explored. Participants talked about the need and effectiveness of mental health services and the misconceptions regarding these services.

#### Subtheme: Parents’ Perception of the Need to Access Mental Health Services

Conceptions about the need for mental health support varied across parents. Almost half of parents stated that mental health services are not needed for their children and perceived their child’s ASD symptoms as not severe enough to require mental health services, and their child is happy. An identified mindset is that the child is alone and needs to be merged with other children to overcome mental health issues. One shared perception was that ASD is not a mental health problem; it is a developmental delay.

On the other hand, other parents accepted the idea of receiving mental health services. Some parents (n=6) showed willingness to receive mental health services if it is required, based on professionals’ judgment.

I think Autism is not a mental problem, Autism is just a developmental delay and Autism makes every child different, but it doesn’t mean your child is mentally challenged in any way, you just need to address the issues that they have whether it is sensory, whether it is speech, whether they have problems expressing themselves, I don’t see Autism as mental challenge. (Participant. 18)

#### Subtheme: Parents’ Perception of the Effectiveness of the Mental Health Services

In some cases, children diagnosed with ASD were referred to mental health interventions to address issues like anger and resistance to performing daily activities. Overall, parents perceived the mental health consultation as ineffective due to a lack of follow-up and reduced interaction with the child since appointments were one-time appointments, upon referral. Parents stated that weekly follow-up in mental health clinics is essential to see improvement. A few parents perceived this advisory intervention as useful, whereby the mental health burden on parents was reduced. However, applying this advice was challenging, as the child needed time to absorb and accept these changes.

There is no change, no improvement at the end, follow up must be weekly, and it should be daily if needed, if they provide us a center, to give daily sessions for children. (Participant. 7)

#### Subtheme: Parents’ Misconceptions

Some parents had misconceptions about mental health, such as the belief in the need for brain imaging to identify mental health conditions, and whether ASD developed due to overuse of technology devices or from the home environment. Parents seem to have reduced awareness about potential diagnoses of ASD and mental health comorbidities.

For me, mental health is something that I never associated that part, so I never thought about it to be very honest. (Participant. 10)

### Theme 4: Parents’ Perception of the Impact of COVID-19 on ASD Service

Parents showed dissatisfaction with the ASD service during the COVID-19 pandemic. Parents were dissatisfied with online ASD early intervention during COVID-19; they perceived the online service as ineffective and doubted the validity of online diagnosis. COVID-19 contributed to a delay in receiving ASD services, as services were suspended and redesigned during the pandemic. In addition, parents highlighted that cases like ASD should not be left out and must be closely monitored, and because of this gap in the service, the child’s condition got worse over time. Parents indicated that the lockdown during COVID-19 had a negative impact on the child’s case.

I did not find a way out. The parks and malls were closed. He was at the beginning of his awareness to know these things. Places close-up was the reason my son’s case got worse. Corona was a difficult period. All these factors led to decline in his progress but thank God I was trying with him. (Participant. 8)

### Theme 5: Impact of Raising a Child Diagnosed with ASD on Parents

Parents reported that parenting a child diagnosed with ASD had an impact on their personal and mental well-being and had a financial impact.

#### Subtheme: Personal Well-being of Parents of Children Diagnosed with ASD

Child’s behavior outside home is a major challenge for parents, as they are forced to follow the child’s daily activities step by step to ensure protecting him/her. In some cases, mothers would not sleep at night due to the fear of injury that may happen to the child at night, given the child’s intermittent sleeping pattern. According to interviewed parents, the experience of having a child with ASD is very exhausting and requires being fully aware and constantly concentrated, with no time to rest, especially while handling it all with other siblings.

I don’t know what hurts him at night, I don’t sleep, just because I fear that something would happen to him at night. (Participant. 9)

#### Subtheme: Mental Well-being of Parents of Children Diagnosed with ASD

The burden highlighted by parents is mainly mental and emotional, especially at the time of diagnosis. Mental health burden stems from the child’s behavior, psychosocial status, and the social stigma behind it. At the time of diagnosis, parents were challenged in terms of the high responsibility behind it, the lack of direction, and feeling lost, and society’s reflection on this diagnosis. Moreover, self-blame was noted among parents, from the point of view that their child's ASD developed due to mis-parenting, neglect, work overload, distracting the child with screen equipment, reduced awareness about ASD, and not doing the genetic testing at an early stage of the child’s age.

The experience of having a child with ASD is more difficult than what the human mind can imagine, it needs you to be alert and awake like in the military, 24 hours 7 days a week, you never have time to rest. At the end we are human beings. (Participant. 7)

#### Subtheme: Financial Impact

Diagnosis with ASD carries a high financial burden on parents. ASD services are expensive, ranging from health interventions, education, support services, and play toys used to support children with ASD, parents stated. As such, parents talked about encountering financial burden to secure a fulfilled life and the demands for their autistic child. Qatari parents waived the need to receive support from the government to secure all ASD services, free of charge or at least at a convenient cost.

The financial side, outside the session that lasts less than an hour could reach QAR 400 – 500, one session is not enough, you find yourself monthly paying not less than QAR 5000-6000, which is out of budget for the majority. (Participant. 7)

## Discussion

In this study, a qualitative approach was employed to dig deeply into parental perceived challenges in raising a child diagnosed with ASD, accessing the early intervention, and receiving mental health services for their children with ASD. The ASD service access journey was explored. The main stages of this journey were the triggers to seeking ASD services, parents’ response to ASD diagnosis, parents’ perception of ASD etiology, and finally accepting Autism.

Previous research showed that parents experienced an emotionally intense journey, ranging from a lack of support, parental initial concerns, and receiving an ASD diagnosis.^24^ Another study showed that at the time between parental initial concerns and diagnosis, parents were in denial, confusion, and were emotionally overwhelmed.^24^ Our study findings are consistent with evidence about the ASD service access journey. The study results call for services mapping efforts to guide parents at the time of diagnosis and intervention, and meet a standardized care pathway.

Found challenges associated with raising a child with ASD were emotional burden, familial and social burdens, distress, and vulnerability.^25^ Another study showed that parenting a child with ASD incurred grief and guilt reactions to ASD diagnosis, burden of care, by which physical and emotional exhaustion are encountered, concerns about the quality of available services, financial burdens, and impact on family relationships and social life.^26^ Obviously, evidence from the literature shows consistency with our study findings.

Exploring parental perception of ASD early intervention revealed the issues of delay in receiving ASD service and other challenges related to the service design, program intensity, and duration. A systematic review reported that parents spend a lengthy period between their initial concerns and receiving professional help, as the process of obtaining an ASD diagnosis was exhaustive and complicated.^24^ Another study showed parental-negative experiences with ASD service at the system level, including difficulty in service access and follow-up, and restriction of care based on specific needs.^14^

Our study shows that parental attention to the mental health of the child diagnosed with ASD is minimal. A few children with ASD were referred to mental health services, and very few parents found this intervention useful. The reduced awareness about the child’s mental health could be explained by healthcare providers’ neglect. As a result, the diagnosis of a mental health condition is missed, due to focusing on the management of ASD symptoms alone rather than managing the case comprehensively. A study showed that parents of children diagnosed with ASD saw a positive impact on their child as a result of the mental health intervention.^27^ In contrast, our study findings showed that, few of those who referred to mental health services found the service useful and were challenged in applying the received consultation with their child.

In alignment with our study findings, a study shows that there is poor access and utilization of evidence-based behavior therapy and mental health support services.^28^ Our study findings show that knowledge and awareness about mental health service needs are limited. Further research about mental health among individuals with ASD is needed to enhance awareness and capacity building in clinical settings.

COVID-19 pandemic harmed the provided service for children diagnosed with ASD and their families, in terms of receiving ASD early intervention on time. Alhuzimi reported that parental stress and emotional well-being were impacted due to barriers in service utilization for ASD children during the pandemic.^29^ This shows consistency with our study findings, as parents in our study indicated that their children were “left out” during the pandemic, and the child’s condition was getting poorer over time since there was difficulty in accessing the service. In our study, the impact of ASD on parents was mainly about the personal and mental well-being of parents of children diagnosed with ASD, and the financial impact. The impact of ASD may include increased parental stress, mental and physical problems, financial burden, high rates of divorce, and affect the overall family well-being.^30^ Moreover, parents of children with ASD may experience high levels of psychological distress and attachment-related anxiety.^31^ This shows consistency with the result of this study.

### Implications for Practice and Research

The findings of this study fill the gap in the literature about parental perceived challenges in raising a child with ASD including access to early intervention for children diagnosed with ASD in Qatar. The study findings guide stakeholders at the policy level and health care professionals working with children with ASD and their families in developing standards of care for ASD service in the governmental facilities in Qatar. Key stakeholders should consider parental perceived challenges in designing support services in different sectors, including community, education, and healthcare. The mental health aspect of childcare should be considered in clinical practice, including diagnosis, intervention, supplementation, and parent support, tailored to each child’s needs. Research addressing the needs of children diagnosed with ASD and their families in a larger sample size is required to have more generalizable findings. Future contextual research is needed to understand the barriers to seeking mental health services among individuals and parents raising children diagnosed with ASD. Across the reported findings in this study, a different set of recommendations was commonly stated among parents to improve ASD services overall. This set of recommendations was to improve services within the health care system, improve services in education settings, offer mental health services for parents of children diagnosed with ASD, and enhance community support services. National stakeholders should work collaboratively to secure ASD services across different domains to support individuals with ASD and their families.

## Strengths and Limitations

The study has several strengths: 1) using the governmental hospital as setting is a window of opportunity to capture rich data from different population makeup in Qatar, socioeconomic statuses, and backgrounds, 2) applying qualitative approach provides a thick description of parents’ experiences, a clear and deep understanding of the study phenomenon, 3) applying triangulation in the analysis process to enhance the credibility of our research, in which the PI and Co-PIs involved in the project worked independently to analyze the data to help in the verification process.

On the other hand, the study may be prone to information bias, as interviewees were asked about their past experiences, and recall bias may be present. Another source of information bias is the researcher's profession in the ASD field. To lessen the effect of such biases, more than one researcher was involved in the transcription, analysis, and interpretation processes, and the researcher’s experience, biases, and values were set aside. We ensured being open to learning about the shared experiences as viewed by parents.

## Conclusion

The study findings were mainly about challenges of raising a child diagnosed with ASD, parental perception of existing ASD services, and parental perception of the mental health services provided for their children. Findings of this study emphasize the importance of stakeholders’ roles in designing ASD services, based on the needs of children with ASD and their families. Health professionals should consider the identified parental needs highlighted in this research. National multi-sectoral efforts are needed to provide appropriate guidance and service mapping for individuals diagnosed with ASD and their families.

## Data Availability

The data used and/or analysed during the current study are available from the corresponding author on reasonable request.

## Abbreviations

ASD, Autism Spectrum Disorder; WHO, World Health Organization; ABA, Applied-Behavior Analysis; EB, EarlyBird; COVID-19, Coronavirus disease of 2019; QAR, Qatari Riyal.
